# Molecular Mechanisms Governing “Hair-Trigger” Induction of Shiga Toxin-Encoding Prophages

**DOI:** 10.3390/v10050228

**Published:** 2018-04-29

**Authors:** Dolonchapa Chakraborty, Eric Clark, Steven A. Mauro, Gerald B. Koudelka

**Affiliations:** 1 Department of Biological Sciences, University at Buffalo, Buffalo, NY 14260, USA; dolon8619@gmail.com; 2 Department of Biology, Mercyhurst University, Erie, PA 16546, USA; eclark5289@gmail.com; 3 Department of Biology, Gannon University, Erie, PA 16541, USA; mauro002@gannon.edu

**Keywords:** bacteriophage, Shiga toxin, DNA binding, transcription

## Abstract

Shiga toxin (Stx)-encoding *E. coli* (STEC) strains are responsible for sporadic outbreaks of food poisoning dating to 1982, when the first STEC strain, *E. coli* O157:H7, was isolated. Regardless of STEC serotype, the primary symptoms of STEC infections are caused by Stx that is synthesized from genes resident on lambdoid prophage present in STEC. Despite similar etiology, the severity of STEC-mediated disease varies by outbreak. However, it is unclear what modulates the severity of STEC-mediated disease. Stx production and release is controlled by lytic growth of the Stx-encoding bacteriophage, which in turn, is controlled by the phage repressor. Here, we confirm our earlier suggestion that the higher spontaneous induction frequency of Stx-encoding prophage is a consequence, in part, of lower intracellular repressor levels in STEC strains versus non-STEC strains. We also show that this lowered intracellular repressor concentration is a consequence of the utilization of alternative binding/regulatory strategies by the phage repressor. We suggest that a higher spontaneous induction frequency would lead to increased virulence.

## 1. Introduction

Shiga toxin (Stx) is an essential virulence factor in human disease that is caused by infection with Stx-encoding *E. coli* (STEC) [1,2,3]. STEC are an important and increasing global public health concern, as they are the causative agents for various clinical symptoms including severe bloody diarrheal syndrome, hemorrhagic colitis, neurological disorders, and hemolytic-uremic syndrome (HUS) [4]. The genes that encode Stx in STEC are exclusively associated with active or cryptic lambdoid prophages. The family of λ-like Stx-encoding phage is large, diverse, and ubiquitously distributed among a wide variety of *E. coli* serotypes [5].

The *stx* genes in phage that encode Stx 2 are located downstream from a promoter that is active only during phage lytic growth, meaning that Stx 2 is produced only during phage lytic growth [6,7,8,9,10,11]. As a result of its position in the phage lytic cascade, synthesis of Stx 2 is ultimately regulated by the DNA binding and transcriptional regulatory activities of the bacteriophage repressor. Stx is not exported by any bacterial secretory machinery [12,13]. Instead, the release of this exotoxin from the bacteria depends on phage genes that cause bacterial lysis [14,15], the expression of which are also ultimately regulated by the bacteriophage repressor. Repressor negatively regulates Stx 2 synthesis and release by preventing expression of the viral genes necessary for lytic growth. Thus, the DNA binding and transcriptional regulatory activities of the bacteriophage repressor are vital regulators of STEC virulence.

Repressor regulates phage growth and Stx 2 expression by binding to the operator regions O_R_ and O_L_, and repressing transcription initiation at two early promoters, P_R_ and P_L_, respectively. Concomitantly, repressor binding at O_R_ stimulates transcription from promoter P_RM_, enhancing transcription of the repressor-encoding *cI* gene, thereby stabilizing the lysogenic state of the prophage. As a result of repressor’s DNA binding and regulatory activities, in a prophage, the promoters for lytic genes are totally repressed by repressor binding, meaning that the *stx* genes reside harmlessly within the lysogenic bacterial host.

Induction of the STEC lambdoid prophage, and the consequent production and release of phage and Stx, require that repressor’s gene regulatory activities be inactivated. Although several mechanisms for inducing lambdoid phages have been identified [16,17,18], the best understood mechanism of induction involves RecA, which is central to the bacterial SOS response. The interaction of repressor with activated RecA (RecA*) stimulates the intrinsic auto-proteolytic activity of the repressor. Repressor auto-cleavage dramatically reduces its affinity for its DNA binding sites, thereby de-repressing the genes needed for phage lytic growth [19,20]. Regardless of the induction mechanism, this process concomitantly allows expression of both the genes encoding *stx*, and those needed for its release [21,22]. Since Stx has been implicated in causing the symptoms in STEC-mediated disease, an increased rate of induction would lead to higher levels of Stx, thereby resulting in a more virulent infection.

Despite the similarities in the mechanism of lysogen maintenance and induction among all lambdoid prophages, several Stx-encoding prophages spontaneously induce at a much higher frequency than do related, non Stx-encoding lambdoid prophages [23]. This property is known as “hair-trigger” induction. Hair-trigger induction has been proposed to contribute to both the virulence and dispersion of the Stx-encoding phage [24]. Since lambdoid prophage induction is a direct outcome of repressor auto-cleavage, and repressor auto-cleavage is stimulated by RecA, Livny and Friedman [23] suggested a lower concentration of RecA* may be needed to induce cleavage of repressor in this class of phage.

Given the central role of the repressor in governing prophage induction, we have been probing the activities of the repressors of Stx-encoding phages and comparing them with that of the repressors from phages that do not encode Stx. In particular, we are examining the Stx 2-encoding prophages 933W [25] and BAA2326 [26], both of which undergo spontaneous induction at higher frequencies than do non-Stx-encoding prophages, 434 and λ [27]. Since the higher spontaneous induction frequencies of these Stx encoding phages are seen both in wild-type *E. coli* strains and in *rec*A mutant strains, the observed increased induction frequency of these prophages is at least partially independent of RecA*-stimulated repressor auto-cleavage [27]. Our earlier results suggested that the activity of P_RM_, the promoter that directs transcription of the gene that encodes repressor, is lower in Stx-encoding lysogens [27,28]. We suggest that the increased induction frequency of the Stx-encoding prophage may be due, at least in part, to a lower amount of repressor present in the lysogenic cell. Although this suggestion is consistent with the predictions of Livny and Friedman [23], the underlying mechanism(s) that lead to lower intracellular repressor concentrations in Stx-encoding prophages has not yet been determined. Previous studies indicated that the lower amount of repressor-encoding mRNA synthesized from P_RM_ in another Stx-encoding prophage, 933W, is a result of its repressor partially occupying O_R_3, and thereby repressing transcription from 933W P_RM_ [28]. Consequently, we hypothesize that increased spontaneous induction as a consequence of partial O_R_3 occupancy and P_RM_ is a feature of repressor gene regulation in Stx-encoding phages.

To test this hypothesis and to elucidate these mechanisms, we examined the DNA binding and transcriptional regulatory activities characteristics of BAA2326 repressor, and compared them with the Stx-encoding phage 933W and non Stx-encoding phages, 434 and λ. Knowing the underlying mechanisms that cause an increased susceptibility to induction will lead to a better understanding of the features of Stx-encoding phages that underlie their reduced stability/increased inducibility. Knowledge of these features should provide insight into how these phages evolved to become highly prevalent, and why they became highly successful in causing human disease.

## 2. Materials and Methods

### 2.1. Bacterial Strains, Bacteriophages, and DNA

*E. coli* strains BAA2326 (containing phage BAA2326), EDL 933 (containing phage 933W), K802, and MG1655 were obtained from the American Type Culture Collection (Manassas, VA, USA). Bacterial cultures were grown with agitation in Luria Broth (LB) at 37 °C. Phages were obtained by induction of the respective host strains with mitomycin C (1 µg/mL). λ and 434 were obtained from our collection. BAA2326 and 933W lysogens were constructed as described previously [29].

All plasmids were propagated in *E*. *coli* strain K802. BAA2326 repressor was purified from the *E*. *coli* strain BL21(DE3) pLysS (Novagen, Madison, WI, USA) bearing a plasmid that directs its overexpression. Primers for site-directed mutagenesis and making other templates for DNA binding experiments were obtained from Integrated DNA Technologies (Coralville, IA, USA).

### 2.2. Quantitation of Intracellular Repressor Concentration

Cultures of MG1655 lysogens containing BAA2326, 933W, λ, or 434 were grown overnight at 37 °C. The following day, the cultures were diluted 50-fold and grown to mid-log phase and cells harvested by centrifugation (see below). Harvested cells were dispersed in sterile distilled water and plated to quantify the number of bacteria. In parallel, repressor levels in 933W, λ, and 434 lysogens were measured using quantitative western blots, and in BAA2326 lysogens using quantitative dotblots. Polyclonal antibodies to the λ repressor, which also detects BAA2326 repressor, were a generous gift from Dr. Sankar Adhya (Center for Cancer Research, National Cancer Institute, Bethesda, MD, USA). Antibodies to the 434 and 933W repressors were from our collection [30].

### 2.3. Quantitative Western Blots

Cells from mid-log phase cultures of 933W, λ, and 434 lysogens were harvested by centrifugation at 2000× *g* for 5 min. The supernatant was discarded and the pellet was dispersed in Lammeli buffer (4% SDS, 20% glycerol, 10% β-mercaptoethanol, 120 mM Tris-HCl (pH 6.8), 0.02% bromophenol blue). The proteins were fractionated by SDS-PAGE and transferred to Polyvinylidene Fluoride (PVDF) membrane (GE Lifesciences, Piscataway, NJ, USA) by electroblotting. The blots were incubated with blocking buffer (5% Bovine Serum Albumin (BSA) in PBST (PBS with 0.05% Tween-20) for 1 h at room temperature. Blots were then incubated with either 933W (Figure S1B), 434 (Figure S1C), or λ (Figure S1D) rabbit primary antibodies in blocking buffer for 1 h at room temperature. Blots were washed twice with PBST and incubated with horseradish peroxidase-conjugated secondary antibody against rabbit IgG (Thermo Scientific, Waltham, MA, USA), and were incubated for 1 h at room temperature. Blots were again washed twice each with PBST and distilled water. Proteins were visualized using Pierce™ ECL Western Blotting Substrate (Thermo Scientific, Waltham, MA, USA), and visualized using ChemiDoc™ XRS+ System (Bio-Rad, Hercules, CA, USA). Signal intensity was measured using the ImageLab software (Bio-Rad, Hercules, CA, USA).

### 2.4. Quantitative Dotblots

Due to the low intracellular concentration of BAA2326 repressor, we were unable to load sufficient cell extract on an SDS-PAGE gel to detect it by western blot. Thus we quantified the amount of this protein on a dotblot. For this method, cells from mid-log phase cultures of BAA2326 lysogens were harvested by centrifugation at 4000× *g*, and the pellet was dispersed in suspension buffer (0.1 mM dithiothreitol, 6 mM MgCl_2_, 40 mM Tris-HCl (pH 7.5) containing protease inhibitors (5 μg/mL of leupeptin/mL, 50 μg/mL of benzamidine/mL, 10 U/mL of aprotinin, 5 μg/mL of pepstatin/mL, and 5 μg/mL of TPCK (tosylsulfonyl phenylalanyl chloromethyl ketone)). Subsequently, 0.5 mg/mL DNaseI (Promega, Madison, WI, USA) and 0.5 mg/mL RNase (Thermo scientific, Waltham, MA, USA) were added as per manufacturer’s instructions, and incubated at 37 °C for 2 h. Samples were then spotted on a PVDF membrane (GE Lifesciences, Piscataway, NJ, USA) and the membrane was allowed to dry (Figure S1A). Membranes were treated and proteins visualized, as described above.

To precisely determine the amounts of the various repressors in each sample, we transferred or blotted serial dilutions of the respective purified repressors (see [31,32,33] for purification methods) onto the same membrane as the experimental sample (Figure S1), measured the intensity of antibody reactivity, and used this data to construct a standard curve. The concentration of the repressor in each sample was then extrapolated using these plots. The number of repressor monomers per cell were then calculated using measured cell numbers. The number of repressor monomers present per cell are presented as means ± SD. Each measurement consisted of five biological replicates, each with four technical replicates. Statistical comparisons were made by Student’s *t*-test, and *p* values of <0.01 were considered to be significant.

### 2.5. Purification of BAA2326 Repressor

An overnight culture of BL21(DE3) pLysS cells bearing a derivative of the pET15b that directs the overexpression of BAA2326 repressor was diluted 50-fold into 3 L of pre-warmed LB supplemented with 100 μg/mL of ampicillin. After 2 h of growth at 37 °C, production of the repressor was induced by adding 0.5 mM IPTG (isopropyl-β-d-thiogalactopyranoside) to the cultures. After an additional 3 h of growth at 37 °C, the induced cells were harvested by centrifugation at 10,000× *g* for 10 min. The cell pellet was re-suspended in 25 mL of lysis buffer (10 mM Tris pH 8; 500 mM NaCl) and protease inhibitors. All subsequent procedures were performed at 4 °C. Cells were lysed in a French press, and the resulting lysate was diluted to 100 mL with a lysis buffer. Cellular debris was removed from the diluted lysate by centrifugation at 10,000× *g* for 80 min, and the supernatant was equilibrated with lysis buffer. The protein solution was loaded onto an Nitrilotriacetic acid (NTA) resin (Thermo-Fisher, Waltham, MA, USA), and washed with three column volumes of lysis buffer. Subsequently, the column was washed with two column volumes of lysis buffer plus 10 mM imidazole followed by lysis buffer plus 50 mM imidazole. Repressor was eluted from the column using a 50–400 mM imidazole gradient. Repressor-containing fractions eluted from this column were pooled, concentrated, and dialyzed against 50 mM sodium phosphate, pH 6.8 plus 200 mM NaCl. The pooled fractions were concentrated, and glycerol was added to a final concentration of 20%. The repressor was flash frozen and stored at −80 °C. As judged by silver staining of a SDS-PAGE gel of the purified repressor, the repressor is >98% pure. The overall yield of BAA2326 repressor was ∼10 mg/L of bacterial culture.

### 2.6. Electrophoresis Mobility Shift Assays (EMSAs)

DNA containing the putative O_R_ between the putative ‘*cro*’ and *cI* genes was amplified by PCR, using the primers 5′CCCGGCATGAATTCGGGGTTAACGGTTTCTTTTTCATG3′ and 5′GGGCCATAAGCTTGCCAGGGTCTCTTTTTTCATCGCC3′. Following gel purification of the PCR products, the DNA fragments were radioactively labeled at their 5′ ends by incubating the DNA with γ-[32P]-ATP (6000 Ci/mmol) (Perkin-Elmer, Boston, MA, USA) in the presence of T4 polynucleotide kinase (Epicentre, Inc. Madison, WI, USA) at 37 °C for 1 h. After removing unincorporated label, the labeled DNA was incubated with the specified concentrations of BAA2326 repressor in binding buffer (10 mM Tris pH 8.0, 50 mM NaCl, 1 mM EDTA, 10% glycerol, 100 µg/mL BSA, 1 mM DTT) for 5 min at 4 °C. The protein-DNA complexes were resolved on 5% polyacrylamide gels at 25 °C in Tris borate EDTA (TBE—89 mM Tris (pH 8.9), 89 mM borate, 1 mM EDTA) and dried. The amounts of protein-DNA complexes present were quantified using a Storm imager (GE Lifesciences, Piscataway, NJ, USA).

For some experiments, complementary oligonucleotides encoding the sequence of individual BAA2326 repressor binding sites were obtained from Integrated DNA Technologies (Coralville, IA, USA). The sequences of the sites in the 5′→3′direction are:

- O_R_1
  5′GCGAATTCACTAAAGCACTTGCATTTAATAACTGTGCGTAATGATATGCCATAATAACTAAGAAGCTTCC3′
- O_R_2
  5′GCGAATTCACTAAAGCACTTGCAATATGTAATGAAATATCTGCGGTGTTGACATTCCATAATAACTAAGAAGCTTCC3′
- O_R_3
  5′GCGAATTCACTAAAGCACTTGCAATATCACCTTTGGTTATATGTAATGCCATAATAACTAAGAAGCTTCC3′

To prepare these DNAs for use in EMSA, equivalent amounts of each pair of the complementary strands were mixed, heated to 95 °C for 5 min, and slowly cooled over 2–3 h to anneal the two strands. Double-stranded DNA was purified from an acrylamide gel and radioactively labeled as described above.

Values of the dissociation constant were determined by nonlinear squares fitting of the EMSA data to a hyperbolic equation using Prism 4.0 software (GraphPad Software Inc., LaJolla, CA, USA). Each dissociation constant was determined from at least five replicate measurements (Table 1 and Figure S2).

### 2.7. DNaseI Footprinting

The templates that were used for EMSAs were also used for DNaseI footprinting. To provide a uniquely labeled end, the radiolabel from one strand of the DNA prepared as described above was removed by cleavage at an EcoRI site located near one end of the DNA. Following phenol/chloroform/isoamyl alcohol extraction and ethanol precipitation, template DNA was incubated without or with the BAA2326 repressor in a binding buffer (10 mM Tris pH 8.0, 50 mM NaCl, 1 mM MgCl_2_, 100 µg/mL BSA, 5 µg/mL chicken blood DNA, 1 mM DTT) for 5 min at 25 °C, prior to the addition of sufficient DNaseI to generate, on average, one cleavage per DNA molecule in 5 min of additional incubation. The cleavage reactions were terminated by precipitation with ethanol, dehydrated with sec-butanol, and the precipitated DNA was collected by centrifugation. The DNA was dissolved in 90% formamide solution containing tracking dyes. The reaction products, along with chemical sequencing reactions [34] derived from the same templates, were resolved on 6% acrylamide gels containing 7 M urea in TBE. Cleavage products were visualized using a Storm imager (GE Lifesciences, Piscataway, NJ, USA).

### 2.8. Single Round In Vitro Transcription

Transcription reactions were performed essentially as described previously [35], using templates prepared by PCR with primers that amplify wild-type BAA2326 O_R_ or O_R_ regions bearing mutations in either O_R_2 (O_R_2^−^ 5′GTAATGAAATATCTGCGGGCTTGACATTTTAATAACTGTGCG3′) or O_R_3 (O_R_3^−^ 5′GGCGTCTATAAAGTAATGTATATGGGTTTCCACTATACCATAC3′). Increasing amounts of BAA2326 repressor were incubated with 8 nM template DNA for 5 min at 25 °C in transcription buffer (0.2 M Tris-HCl (pH 7.5), 75 mM KCl, 50 mM MgCl_2_, 0.05% Triton X-100, 1 mM dithiothreitol (DTT)). *E*. *coli* RNA polymerase (2 mg/mL) was added and allowed to form open complexes at 37 °C for 15 min. Single-round transcription was then initiated by adding a nucleoside triphosphate mix (2.5 mM each for ATP, CTP, and GTP, 0.5 mM UTP, and α-[32P]-UTP, and 1 mM heparin), and the reaction mixture was incubated at 37 °C for 15 min. Transcription was stopped by adding a formamide containing dye (1% bromophenol blue, 1% xylene cyanol, 20X TBE, formamide), and heated to 95 °C for 4 min. Products were then separated on a 6% acrylamide–7 M urea gel in TBE. Radiolabeled products were visualized and quantified using a Storm imager (GE Lifesciences, Piscataway, NJ, USA).

### 2.9. Primer Extension Assay

BAA2326 lysogens in MG1655 were grown to mid-log phase at 37 °C in LB. Total RNA was extracted from 25 mL of cells using hot phenol [36]. Residual genomic DNA was removed by treatment with RNase-free DNaseI, following manufacturer’s instructions (Promega, Madison, WI, USA). The start site of P_RM_ transcription was determined using the primer sequence 5′CCAGCTTGTACGCAGGAGAG3′ (Figure S3). The primer was radioactively labeled at its 5′ end by incubating with γ-[32P]-ATP (6000 Ci/mmol) (Perkin-Elmer, Boston, MA, USA) in the presence of T4 polynucleotide kinase (Epicentre, Inc., Madison, WI, USA) for 30 min. DNaseI treated total RNA was incubated with the ^32^P-labeled primer at 80 °C for 5 min, followed by cooling to 45 °C to allow primer annealing. Following this, a buffer (50 mM Tris-HCl (pH 8.3), 50 mM KCl, 4 mM MgCl_2_, 10 mM DTT), 2.5 mM dNTPs, and recombinant M-MuLV reverse transcriptase (Thermo Scientific, Waltham, MA, USA) were added and incubated at 45 °C for 30 min. Formamide dye was then added and the mixture was incubated at 95 °C for 5 min to terminate the reaction. Control experiments were performed without reverse transcriptase (RT control) and without RNA (no template control). To rule out non-specific primer binding, 933W RNA was used as control RNA The products of all reactions were separated on an 8% PAGE-urea gel alongside a ^32^P-labeled DNA ladder (Thermo Scientific, Waltham, MA, USA) of known molecular weights. The products of the reaction were visualized by autoradiography using a Storm imager.

## 3. Results

### 3.1. The BAA2326 Repressor Binds to Three Sites in the O_R_

To begin to test the hypothesis that decreased repressor intracellular repressor concentration is a consequence of partial O_R_3 occupancy and P_RM_, and that this gene regulation strategy is common among Stx-encoding phages, we first examined the DNA binding and transcriptional regulatory activities of the BAA2326 repressor.

**Figure 1:**
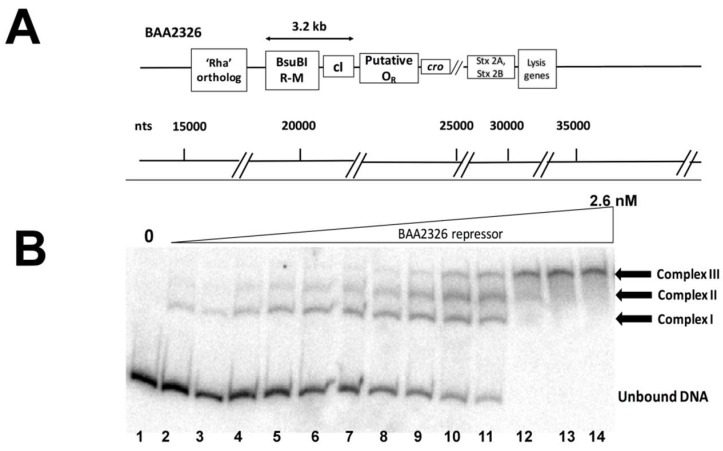
BAA2326 repressor binds to three binding sites in BAA2326 O_R_. (**A**) Proposed organization of genes and putative repressor operator site within the BAA2326 phage immunity region. The separation between the regions typically occupied by the left and right operators (O_L_ and O_R_) is by 3.2 kb. (**B**) A radioactively labeled DNA fragment containing wild-type BAA2326 O_R_ was incubated with increasing concentrations of the BAA2326 repressor. The concentration of the repressor was increased in 1.5-fold steps starting at 0.02 nM. Shown is a native gel of the resulting complexes visualized by phosphorimaging. The positions of the three complexes and the unbound DNA are indicated.

The BAA2326 repressor is closely related to the λ repressor (61.2% identical, 77% similar). In λ*, cI* is located between O_R_ on the right side, and a rexA-rexB exclusion system followed by O_L_ and the gene encoding the *N* anti-termination protein on the left side. However, inspection of the BAA2326 genomic sequence suggests that the sequence of the putative O_R_ region of BAA2326 differs significantly from that of λ. Moreover, there is no readily identifiable O_L_ or *N* gene to the left of the *c*I gene. Instead, genes encoding a *Bsu*BI restriction-modification system are located to the left of *c*I. These genes are followed by ~1 kb of DNA that does not appear to encode any polypeptides (Figure 1A). Therefore, as a first step in understanding how BAA2326 repressor regulates its own expression, we sought to identify the repressor binding sites in its “putative” O_R_ region.

For these experiments, we amplified and radiolabeled the DNA in the region between *cI* and ‘*cro*’ (Figure 1A), incubated it with increasing concentrations of purified BAA2326 repressor, and visualized the repressor-DNA complexes by EMSA. When increasing concentrations of repressor are added to the putative O_R_ DNA, three complexes of decreasing mobility form sequentially (Figure 1B): complex I forms in the presence of ~0.02 nM BAA2326 repressor; complexes II & III appear at nearly identical protein concentrations (~0.05 nM). All of the O_R_ DNA is shifted into complex III by 0.75 nM repressor. Together, these observations suggest that the putative BAA2326 O_R_ contains three repressor binding sites: one with higher affinity and two others with somewhat lower, but nearly identical affinities.

**Figure 2:**
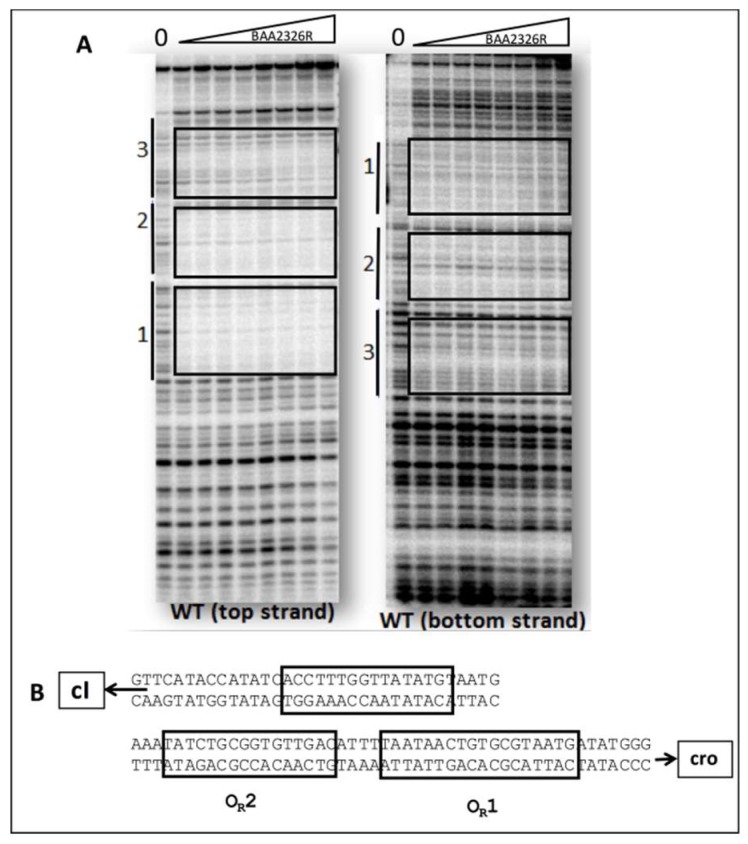
DNaseI footprinting analysis of BAA2326 repressor-O_R_ complexes. (**A**) DNaseI footprinting of complexes between BAA2326 repressor and BAA2326 O_R_. DNA templates containing radioactively labeled BAA2326 O_R_ were partially digested by DNaseI in the presence of increasing amounts of the BAA2326 repressor. The leftmost lane of each panel shows the DNaseI cleavage pattern of the DNA in the absence of added repressor. In lanes 2 to 9, repressor concentrations were increased in 1.5-fold steps starting at 0.03 nM protein. The left panel is the top strand and the right panel is the bottom strand. (**B**) Positions of BAA2326 binding sites within BAA2326 O_R_. The positions of the binding sites (boxed) were identified from the footprinted regions run next to Maxam Gilbert sequencing reactions.

We used DNaseI footprinting to directly visualize the regions bound by the repressor, and thereby confirm the suggestion that BAA2326 repressor binds to three sites in its putative O_R_. Incubating BAA2326 O_R_ DNA with increasing concentrations of BAA2326 repressor results in progressive protection of three individual regions of the DNA from DNaseI cleavage (Figure 2A). We quantified the DNAse I footprinting data to determine repressor’s affinity for the regions. Consistent with the EMSA data, BAA2326 repressor displayed a hierarchy of affinities for the binding sites in O_R_. It binds with highest affinity (K_D_^App^ 28.55 nM) to the site that is proximal to the *cro* gene. By convention, we designated this site as O_R_1. At higher repressor concentrations, 44.01 and 59.07 nM respectively, the protein subsequently binds to two other sites, which we designated as O_R_2 and O_R_3. The absolute values of the apparent dissociation constants (K_D_^App^) determined from DNAse I footprinting differ from those determined by EMSA. We attribute these differences to the different conditions (e.g., buffer, incubation conditions, DNA concentration). Nonetheless, and most importantly, the ratio of binding affinities for the specific sites in O_R_ obtained by the two methods are essentially identical. Thus, complex I in Figure 1 contains BAA2326 repressor bound to O_R_1, complex II contains BAA2326 repressor bound to O_R_1 and O_R_2, and complex III contains the repressor bound all three sites in O_R_.

In addition to providing information about the relative affinities of BAA2326 repressor for its binding sites in O_R_, the DNase I footprinting results also allow us to identify the location and putative sequences of the repressor binding sites in BAA2326 O_R_ (Figure 2B):

O_R_1- 5′ATAACTGTGCGTAATG3′
O_R_2- 5′ATATCTGCGGTGTTGAC3′
O_R_3- 5′CCTTTGGTTATATGTAAT3′

Inspection of these sequences indicates that, unlike the operator sites of other phage repressors, the operator sites in BAA2326 O_R_ exhibit weak rotational symmetry (λ O_R_1: TATCACCGCCAGTGGTA; the underlines indicate the rotational symmetric base sequences). Consequently, the sequences of the individual binding sites also seemingly do not show a strong similarity to one another. The weak rotational symmetry and relatively low sequence conservation are unusual for lambdoid phage “CI-type” repressors, and are surprising, given the high degree of sequence similarity between the DNA binding domains of the BAA2326 and λ repressors.

The binding results show that the relative dissociation constants of BAA2326 repressor for its three sites in intact O_R_ are as follows: O_R_1 = 1, O_R_2 = 1.5, and O_R_3 = 2. The values differ from those found with the other well-characterized Stx-encoding phage, 933W (O_R_1 = 1, O_R_2 = 13.6, and O_R_3 = 26.4) [28], and from those seen with non Stx-encoding phages, λ (O_R_1 = 1, O_R_2 = 2, and O_R_3 = 30) [37] and 434 (O_R_1 = 1, O_R_2 = 1, and O_R_3 = 12) [38,39] (Table 1). Nonetheless, consistent with the suggestion that the repressors of Stx-encoding phages partially occupy O_R_3 in a lysogen, we find that in an intact O_R_, the repressors of Stx-encoding phages, BAA2326 and 933W, bind their respective O_R_2 and O_R_3 with similar relative affinities. In contrast, in non Stx-encoding phages, λ and 434, the respective repressors bind their cognate O_R_2 sites in intact O_R_ 10-20-fold more tightly than to their O_R_3 sites [28,37,38] (Table 1).

### 3.2. The BAA2326 Repressor Binds to O_R_1 and O_R_2 Cooperatively

In most lambdoid phages, the short-range cooperative binding of repressor to O_R_1 and O_R_2 allows repressor to bind O_R_1 and O_R_2 at nearly identical concentrations. Although in some lambdoid phages, repressor occupancy of O_R_3 is mediated by long-range cooperative binding between repressors bound to O_L_3 and O_R_3, the ratio of repressor’s affinities for O_R_2 and O_R_3 sites is largely determined by short-range cooperative binding. We previously showed that the repressor of the Stx-encoding 933W phage does *not* bind cooperatively to O_R_1 and O_R_2, and that this phage does not have an O_L_3 site. Thus, in Stx-encoding 933W phage, the relative affinity of repressor for O_R_2 and O_R_3 is determined by the differences in repressor’s intrinsic affinities for these two sites. Having shown that BAA2326 repressor has similar affinities for its O_R_2 and O_R_3 sites in intact O_R_, we wished to determine whether cooperativity or intrinsic affinity differences governs the relative operator affinities of the BAA2326 repressor. To do this, we measured the affinities of BAA2326 repressor for three synthetic oligonucleotides, each containing the sequence of one of the putative operators, O_R_1, O_R_2, or O_R_3, as identified by DNase I footprinting (Figure S2). We then compared these values to those found in intact O_R_. We found that the BAA2326 repressor binds most tightly to its O_R_1 site (K_D_ =0.42 nM), and with ~85-fold and 2-fold lower affinities to the separate O_R_2 and O_R_3 sites, respectively (Table 1). Thus, relative to O_R_1, BAA2326 has a higher affinity for O_R_2 when these two sites are adjacent to each other in intact O_R_ than when they are on separated sites. This observation indicates that BAA2326 repressor binds cooperatively to its O_R_1 and O_R_2 sites in intact O_R_. The strength of the “helping” effect of this cooperativity is similar to that displayed by λ repressor binding to its O_R_1 and O_R_2 sites in intact λ O_R_ [40]. Therefore, the similarity in BAA2326 repressor’s affinities for its O_R_1 and O_R_2 sites is seemingly a result of cooperative repressor binding, and not due to similarity in the intrinsic affinities of the repressor for these two sites, as seen with 933W repressor. Similarly, these observations indicate that short-range cooperativity governs the relative affinities of repressor for O_R_2 and O_R_3.

### 3.3. Transcriptional Regulation at O_R_ by BAA2326 Repressor

The similar repressor affinities for the O_R_2 and O_R_3 sites in Stx-encoding phage, 933W and BAA2326, suggest that the activity of P_RM_ is largely repressed. If correct, this could result in a lower intracellular repressor concentration. Hence, the differences in relative repressor affinities for O_R_2 and O_R_3 between the Stx-encoding and non Stx-encoding phage may help to explain why the Stx-encoding prophage exhibit unstable “hair-trigger” induction whereas the non Stx-encoding phage do not.

**Figure 3:**
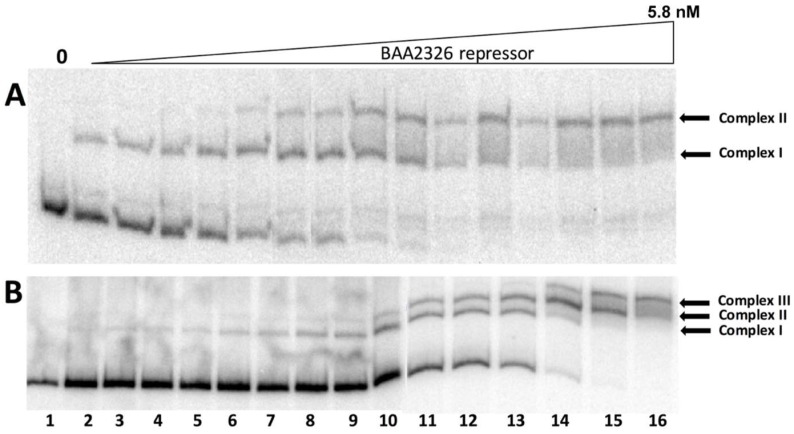
Electrophoretic Mobility Shift Assay (EMSA) analysis of complexes formed between BAA2326 repressor and mutant O_R_ regions. Radioactively labeled BAA2326 O_R_-containing DNA fragments bearing mutations in either. (**A**) BAA2326 O_R_2 or (**B**) BAA2326 O_R_3 were incubated with increasing concentrations of the BAA2326 repressor. The concentration of the repressor was increased in 1.5-fold steps starting at 0.02 nM. Shown are native gels of the resulting complexes visualized by phosphorimaging. The positions of the repressor-DNA complexes are indicated.

To further test the idea that the similar repressor affinities of BAA2326 repressor for O_R_2 and O_R_3 lead to lower P_RM_ activity, we first created O_R_ regions bearing mutations in either O_R_2 or O_R_3, and examined the binding pattern of repressor to these mutant O_R_ regions. The O_R_2 mutation ATATCTGCGGTGTTGAC → ATATCTGCGGGCTTGAC eliminated BAA2326 repressor binding to this site, without seemingly disrupting BAA2326 repressor binding to O_R_1 or O_R_3 (compare Figure 3A with Figure 1B, see also Figure S4). We only observed two repressor-DNA complexes on this template: Complex I which contains repressor bound to O_R_1, and complex II containing repressor bound to O_R_1 and O_R_2. The template bearing the O_R_3^−^ mutations ACATATAACCAAAGGT → ACATGGAACCAAAGGT reduced repressor’s affinity for this site by ~15-fold (compare the concentrations needed to form Complex III in Figure 3B with that of Figure 1B). The O_R_3^−^ mutations apparently do not alter the affinity of BAA2326 repressor for O_R_1 and O_R_2.

**Figure 4:**
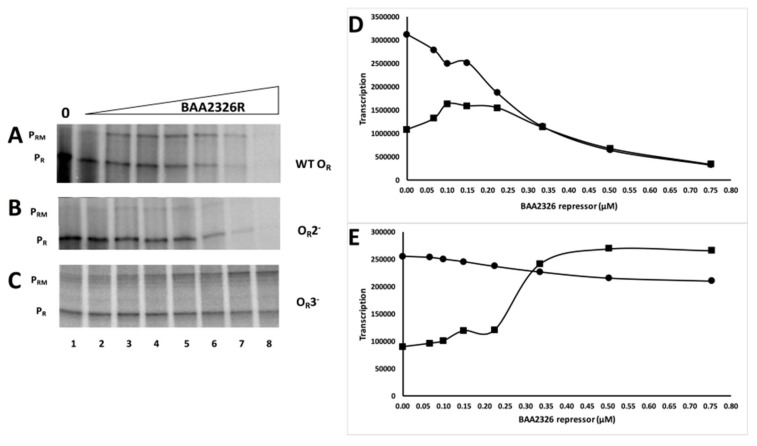
O_R_2 and O_R_3 are required for regulation of P_RM_ transcription by repressor. Shown above is a representative transcription gel. BAA2326 DNA templates containing (**A**) wild-type O_R_, (**B**) O_R_ regions bearing mutations in either O_R_2 (O_R_2^−^) or (**C**) O_R_3 (O_R_3^−^) were transcribed in vitro in the absence of repressor (lane 1) and at repressor concentrations increased in 1.5-fold steps, starting with 66 nM protein. Positions of transcripts initiated from P_R_ and P_RM_ are indicated. The BAA2326 repressor was incubated with DNA template at 25 °C for 10 min, followed by addition of *E. coli* RNA polymerase. The reaction mixture was transferred to 37 °C for 10 min before the transcription reaction was initiated by the addition of nucleotides and heparin. (**D**–**E**) Graphical representation of the amount of P_RM_ and P_R_ transcripts synthesized as a function of BAA2326 repressor concentration from the template bearing wild-type O_R_ (**D**) or templates bearing a mutation in O_R_3 (**E**). The circles represent P_R_ transcription and squares represent P_RM_ transcription.

Having established that these mutations only affect the affinity of the BAA2326 repressor for a single site, we then examined the ability of this protein to control in vitro transcription from P_R_ and P_RM_ on wild-type and the O_R_2^−^ and O_R_3^−^ templates. In wild-type O_R_ in the absence of repressor, only transcripts initiating at the P_R_ promoter are detected (Figure 4A lane 1 and 4D). Adding increasing amounts of BAA2326 repressor to the reaction inhibits transcription from P_R_ and stimulates transcription from P_RM_ (Figure 4A lanes 3–6 and 4D). Adding higher concentrations of repressor represses P_RM_ transcription (Figure 4A lanes 7–8 and 4D). Thus, at higher repressor concentrations, O_R_3 is occupied by repressor, preventing RNA polymerase from initiating transcription from P_RM_. Repressor-mediated repression of P_R_, with concomitant activation of P_RM_, followed by repression of P_RM_ transcription are behaviors common to all known lambdoid bacteriophage repressors. Therefore, with respect to repressor’s transcriptional control of the P_R_ and P_RM_ promoters, BAA2326 behaves as a typical lambdoid phage. Consistent with this conclusion, we found mutations in O_R_2 (O_R_2^−^; ATATCTGCGGTGTTGAC → ATATCTGCGGGCTTGAC) that prevent BAA2326 repressor binding (Figure 3A and Figure S4) to this site and eliminate repressor-stimulated transcription from P_RM_ (Figure 4B). Hence similar to all known typical lambdoid phage repressors, BAA2326 repressor-O_R_2 complex is responsible for the activation of transcription of BAA2326 P_RM_.

Having established the role O_R_2 in P_RM_ activation, we wished to examine the activity of O_R_3 in governing repressor-mediated repression of P_RM_ transcription. In the absence of repressor, on an O_R_3^-^ template only transcripts resulting from RNA polymerase initiating at the P_R_ promoter are detectable (Figure 4C lane 1 and 4E). Adding increasing amounts of repressor to the reaction inhibits transcription from P_R_ and stimulates transcription from P_RM_ (Figure 4C lanes 5–6 and 4E). However, due to the ≥15-fold lower affinity of repressor for the mutant O_R_3, adding 3-fold higher concentrations of repressor than those needed to nearly completely repress P_RM_ transcription on wild-type templates did not repress P_RM_ transcription on the O_R_3^−^ template (Figure 4C lanes 7–8 and 4E). Thus, repressor binding to O_R_3 represses transcription of P_RM_, a function that mirrors that seen with other lambdoid phage repressors. Surprisingly, when compared to its effect on wild-type O_R_, BAA2326 repressor is less effective at repressing P_R_ transcription on the O_R_3^-^ template. We are unsure as to why this is; however, this observation suggests that repressor binding to O_R_3 helps repress P_R_ transcription.

We noticed that a lower maximal amount of transcripts were produced P_RM_ from the wild-type O_R_ template than from the O_R_3^−^ mutant template (Figure 4). This finding suggests that due to partial repressor occupancy of O_R_3 at concentrations needed to fully occupy O_R_2, the BAA2326 repressor is never able to fully activate P_RM_ transcription in wild-type O_R_. To quantify this observation, we compared the maximal amount of transcript synthesized from P_RM_ on wild-type O_R_ with that synthesized from P_RM_ on the O_R_3 mutant template. To facilitate this comparison, we compared the amounts of P_RM_ transcripts with those synthesized from P_R_, which is unaffected by the O_R_3 mutation. On the wild-type O_R_ template, the maximal amount of P_RM_ transcript formed (Figure 4A lane 3 and 4D) is 2-fold less than that from P_R_ (Figure 4A lane 1 and 4D). However, on an O_R_3^−^ template, the maximum transcript from P_RM_ transcription (Figure 4C lane 7 and 4E) is approximately equal to that of P_R_ transcription (Figure 4C lane 1 and 4E). This observation indicates that repressor partially occupies O_R_3 in wild-type O_R_, and thus only weakly activates transcription at P_RM_. This finding is similar to that seen in 933W phage, where complete repression of P_RM_ transcription occurred only with the addition of 4-fold more 933W repressor than is needed to maximally activate this promoter [28]. These findings explain why BAA2326 lysogens synthesize less P_RM_ transcripts than do the non-Stx-encoding phages, λ and 434 [27].

Together with our previous findings [27], the finding that BAA2326 repressor partially occupies O_R_3 and represses the amount of transcripts initiated from P_RM_ suggests that both the Stx-encoding 933W and BAA2326 lysogens may contain lower repressor levels. However, the repressor-encoding mRNAs of BAA2326 and λ [43] do not contain a Shine-Delgarno (SD) sequence, while those of 933W [44] and 434 [45] do. Given these observations, there may not be a direct correlation between transcript levels and protein amounts in these lysogens. Therefore, we directly measured the amount of repressor protein synthesized from the prophage in lysogens.

**Figure 5:**
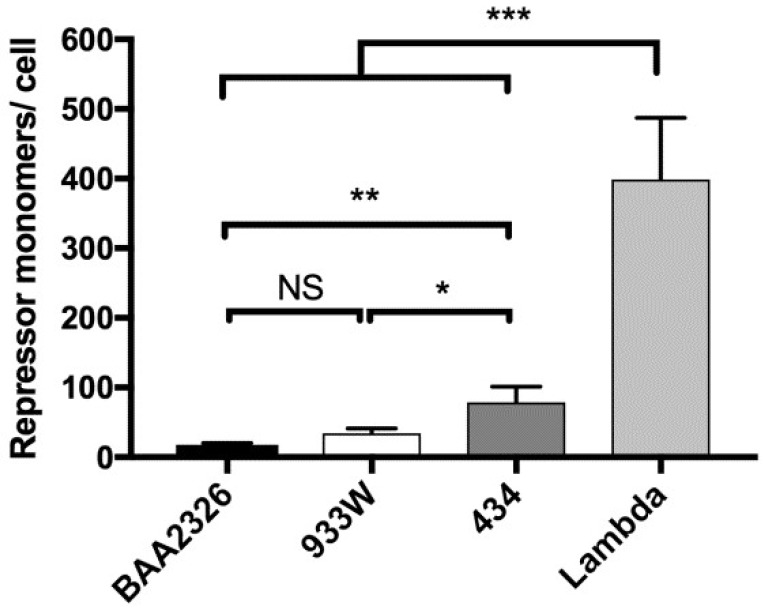
Intracellular repressor levels in MG1655 lysogens. Quantitative Western blot and dotblot were used to determine repressor levels produced from mid-log phase MG1655 lysogenized with BAA2326 (black bars), 933W (white bars), λ (dark gray bars), or 434 (gray bars). Repressor amounts were calculated on a single cell level (see Materials and Methods). The amount of repressor produced by Stx-encoding prophage lysogens (BAA2326 and 933W) is significantly lower than those produced by non Stx-encoding prophage lysogens (λ and 434) (NS: *p* > 0.01, * *p* < 0.05, ** *p* < 0.005, *** *p* < 0.0001)).

Similar to previous measurements [46], we find that lysogens of bacteriophage λ contain 399 repressor molecules per cell, and those of 434 contain 79 repressor molecules per cell (Figure 5). In contrast, but consistent with the low level of P_RM_ transcripts found in BAA2326 and 933W lysogens [27], we find these lysogens contain much lower levels of repressor protein: BAA2326 lysogens contain 18 molecules per cell and 933W lysogens contain 34 molecules per cell (Figure 5). Hence, this observation supports the hypothesis that Stx-encoding lysogens contain lower levels of repressor molecules than do non Stx-encoding lysogens.

These findings suggest a reason why BAA2326 and 933W prophage exhibit “hair-trigger” property, i.e., they only have sufficient repressor to occupy the operators; any minor fluctuation in the intracellular repressor concentration in these lysogens would lead to lower operator occupancy, and consequently, to induction. This finding also suggests that less RecA* would be needed to induce the Stx-encoding prophages than is needed to induce the non Stx-encoding prophages.

## 4. Discussion

“Hair-trigger” induction is apparently a common feature of Stx-encoding prophages [23,27,28]. The results presented here indicate that this increased susceptibility to induction of Stx-encoding prophages is a consequence of a reduced amount of the Stx-encoding phage repressor present in Stx-encoding phage lysogens. While both Stx-encoding and non-encoding phage tightly regulate intracellular repressor concentrations, these two classes of phage employ different overall strategies to do so. In non Stx-encoding prophage (e.g., 434, λ, and P22), P_RM_ transcriptional activity and intracellular repressor levels are controlled by long-distance cooperativity between repressor molecules bound at O_R_ and O_L_ [47]. This long-distance cooperativity facilitates repressor binding to a weak O_R_3. However, the occupancy increase caused by long-distance cooperativity is moderate [48] (~2-fold), and seemingly does not enable repressor to bind to O_R_3 at the same or similar concentrations as it does to O_R_2. Thus, P_RM_ transcript, and therefore, repressor proteins levels in lysogens bearing non-Stx-encoding phage, are not substantially auto-repressed. On the other hand, intrinsic or short-range cooperativity-aided affinities of the repressors of the Stx-encoding phage (e.g., 933W, BAA2326) for their cognate O_R_2 and O_R_3 sites are nearly identical. The similarity in affinity allows them to be bound at nearly identical concentrations. Thus, the repressors simultaneously nearly completely occupy O_R_3, resulting in severe auto-repression of P_RM_ transcription; therefore these lysogens contain a limited amount of repressor.

Despite the similarity in their strategy for regulating intracellular repressor concentrations, EDL933W and BAA2326 prophage use different precise molecular mechanisms to achieve this result. The 933W repressor does not bind cooperatively to O_R_1 and O_R_2, meaning that repressor occupancy of O_R_2 and O_R_3 is solely determined by the differences in 933W repressor’s intrinsic affinities for these two sites. On the other hand, the repressor of BAA2326 does cooperatively bind to its cognate O_R_1 and O_R_2. However, the relative affinity of its O_R_2 is so low, that even with apparently strong “help” in the form of cooperative interactions with an O_R_1-bound repressor, BAA2326 repressor’s O_R_2 affinity only rises to a level that matches that of O_R_3 (Figure 1 and Figure 4, Table 1). Hence, each prophage employs a different binding strategy to regulate repressor levels. Nonetheless, each mechanism causes the lowering of intracellular repressor concentration so that each of these prophages exhibit the property of “hair-trigger” induction.

Given that “hair-trigger” induction is seemingly a common feature of Stx-encoding prophages, the question becomes: what is the benefit of this strategy to these prophages? Insights into the benefits of this strategy come from the realization that most wild-type *E. coli* strains harbor multiple active lambdoid prophages. The time it takes for most prophages to reproduce, mature, and lyse the host are very similar [27,49,50]. Given these similarities, and given that only one phage is generally released from a given cell, we suggest that phages that are able to respond most quickly to an inducing condition will be the most likely “winner” of the race to lyse the cell and be released from it. Hence we suggest that “hair-trigger” induction is an adaptation to the intracellular competition among induced prophages. Regardless of the mechanism used to attain the “hair-trigger” property, we suggest this strategy ultimately leads to an increase in phage fitness. Moreover, when induction is coupled to exotoxin production, “hair-trigger” induction would be expected to cause an increased virulence of the associated host strains. This idea does not suggest that inducers encountered by the Stx-encoding lysogens are not relevant to disease severity. Instead, we suggest that phages employing a hair-trigger induction scheme would be more sensitive to such inducers [27]. Hence the degree to which an Stx-encoding phage utilizes “hair-trigger” property may help to explain the variation in Stx-mediated disease severity from outbreak to outbreak.

## Figures and Tables

**Table 1:** Relative dissociation constants (K_D_) of λ, 434, 933W, and BAA2326 phage repressor DNA complexes formed on individual binding sites in O_R_, when on separate DNA fragments or present in intact O_R_. For BAA2326 repressor, 1 = 4.2 × 10^−10^ M and 2.85 × 10^−8^ M for separate and intact sites respectively. For 933W repressor 1 = 3 × 10^−10^ M and 2.2 × 10^−9^ M for separate and intact sites respectively [28]. For λ repressor, 1 = 1 × 10^−9^ M and 3 × 10^−9^ M for separate and intact sites respectively [40,41]. For 434 repressor 1 = 3.3 × 10^−9^ M and 3 × 10^−8^ M for separate and intact sites respectively [38,42].

Relative KDs								
Phage	λ		434		933W		BAA2326	
Site	Separate Sites	Intact O_R_	Separate Sites	Intact O_R_	Separate Sites	Intact O_R_	Separate Sites	Intact O_R_
O_R_ 1	1	1	1	1	1	1	1	1
O_R_ 2	75	2	14	1	10.3	13.6	83.3	1.5
O_R_ 3	30	30	5.5	12	15	26.4	2.5	2

## Supplementary Materials

The following are available online at http://www.mdpi.com/1999-4915/10/5/228/s1, Figure S1: Quantification of BAA2326, 933W, 434, and λ repressor. Figure S2: Intrinsic affinities of BAA2326 repressor for individual BAA2326 operators, Figure S3: Determining the start of P_RM_, & Figure S4: DNaseI footprinting analysis of BAA2326 repressor-O_R_2^-^ complexes.
