# Supplementary material for: Molecular Mechanisms Governing “Hair-Trigger” Induction of Shiga Toxin-Encoding Prophages
Source: Viruses. 2018 Apr 29;10(5):228. doi: 10.3390/v10050228 (PMC5977221; doi:10.3390/v10050228)
Supplement: Supplementary file 1 [file viruses-10-00228-s001.zip › fiinal supplemental/Binding paper-Supp figure legends NEW-04-29-2018.docx]

**Supplemental Figure 1: Quantification of BAA2326, 933W, 434, and λ repressor.**

(A) Quantitative dotblot was performed on BAA2326 lysogens in MG1655. Lanes 1-11 represent serial dilutions of purified BAA2326 repressor and lanes 12-16 represent BAA2326 samples.

(B) Quantitative western blot was performed on 933W lysogens in MG1655. Lanes 1-4 represent 933W samples, lane 5 was left blank, and lanes 6-9 represent serial dilutions of purified 933W repressor.

(C) Quantitative western blot was performed on 434 lysogens in MG1655. Lanes 1-4 represent 434 samples and lanes 5-7 represent serial dilutions of purified 434 repressor.

(D) Quantitative western blot was performed on λ lysogens in MG1655. Lanes 1-3 represent λ samples, lane 4 was left blank, and lanes 5-8 represent serial dilutions of purified λ repressor.

**Supplemental Figure 2:** **Intrinsic affinities of BAA2326 repressor for individual BAA2326 operators.** A radioactively labeled DNA fragment containing wild-type BAA2326 O_R_1(A), O_R_2 (C), or O_R_3 (E) was incubated with increasing concentrations of the BAA2326 repressor. The concentration of the repressor was increased in 1.5-fold steps starting at 0.03 nM for O_R_1(A), 0.98 nM for O_R_2 (C), or 0.098 nM O_R_3 (E). Shown is a native gel of the resulting complex visualized by phosphorimaging. Binding of BAA2326 repressor to wild-type BAA2326 O_R_1(B), O_R_2 (D), or O_R_3 (F) are indicated as lines representing best nonlinear least-squares fit to the EMSA data based on a hyperbolic equation.

**Supplemental Figure 3: Determining the start of P_RM_**

A) Primer was designed as shown in figure and was radiolabeled and used for primer extension assay. B) Autoradiograph of the primer extension products to determine the start of P_RM_. Primer was radiolabeled (see materials and methods) and incubated with DNase’d RNA extracted from BAA2326 lysogens in MG1655. The corresponding DNA marker is indicated as M.

**Supplemental Figure 4: DNaseI footprinting analysis of BAA2326 repressor-O_R_2^-^ complexes.**

DNaseI footprinting of complexes between BAA2326 repressor and BAA2326 O_R_2^-^. DNA templates containing BAA2326 O_R_2^-^ radioactively labeled were partially digested by DNaseI in the presence of increasing amounts of the BAA2326 repressor. The leftmost lane shows the DNaseI cleavage pattern of the DNA in the absence of added repressor. Repressor concentrations were increased in 1.5-fold steps starting at 0.03 nM protein. The positions of the binding sites (boxed) were identified from Maxam Gilbert sequencing reactions run next to the DNaseI fragments (not shown)

**Supplemental Figure 4: Quantification of BAA2326, 933W, 434, and λ repressor.**

(A) Quantitative dotblot was performed on BAA2326 lysogens in MG1655. Lanes 1-11 represent serial dilutions of purified BAA2326 repressor and lanes 12-16 represent BAA2326 samples.

(B) Quantitative western blot was performed on 933W lysogens in MG1655. Lanes 1-4 represent 933W samples, lane 5 was left blank, and lanes 6-9 represent serial dilutions of purified 933W repressor.

(C) Quantitative western blot was performed on 434 lysogens in MG1655. Lanes 1-4 represent 434 samples and lanes 5-7 represent serial dilutions of purified 434 repressor.

(D) Quantitative western blot was performed on λ lysogens in MG1655. Lanes 1-3 represent λ samples, lane 4 was left blank, and lanes 5-8 represent serial dilutions of purified λ repressor.
