# Supplementary figures and images for: Molecular Mechanisms Governing “Hair-Trigger” Induction of Shiga Toxin-Encoding Prophages
Source: Viruses. 2018 Apr 29;10(5):228. doi: 10.3390/v10050228 (PMC5977221; doi:10.3390/v10050228)

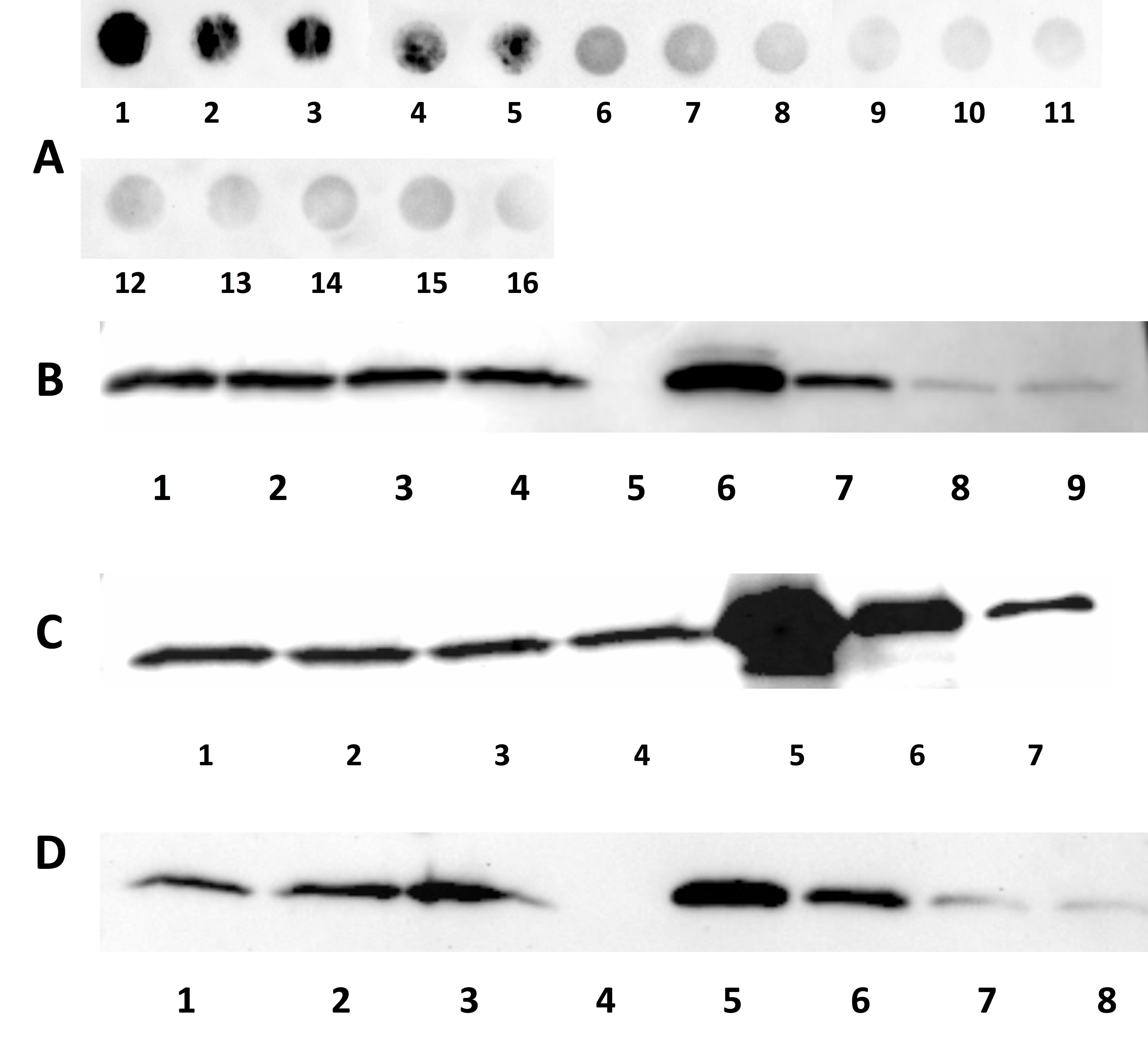

Supplement: Supplementary file 1 [file viruses-10-00228-s001.zip › fiinal supplemental/Figure_S1-Final.tiff]

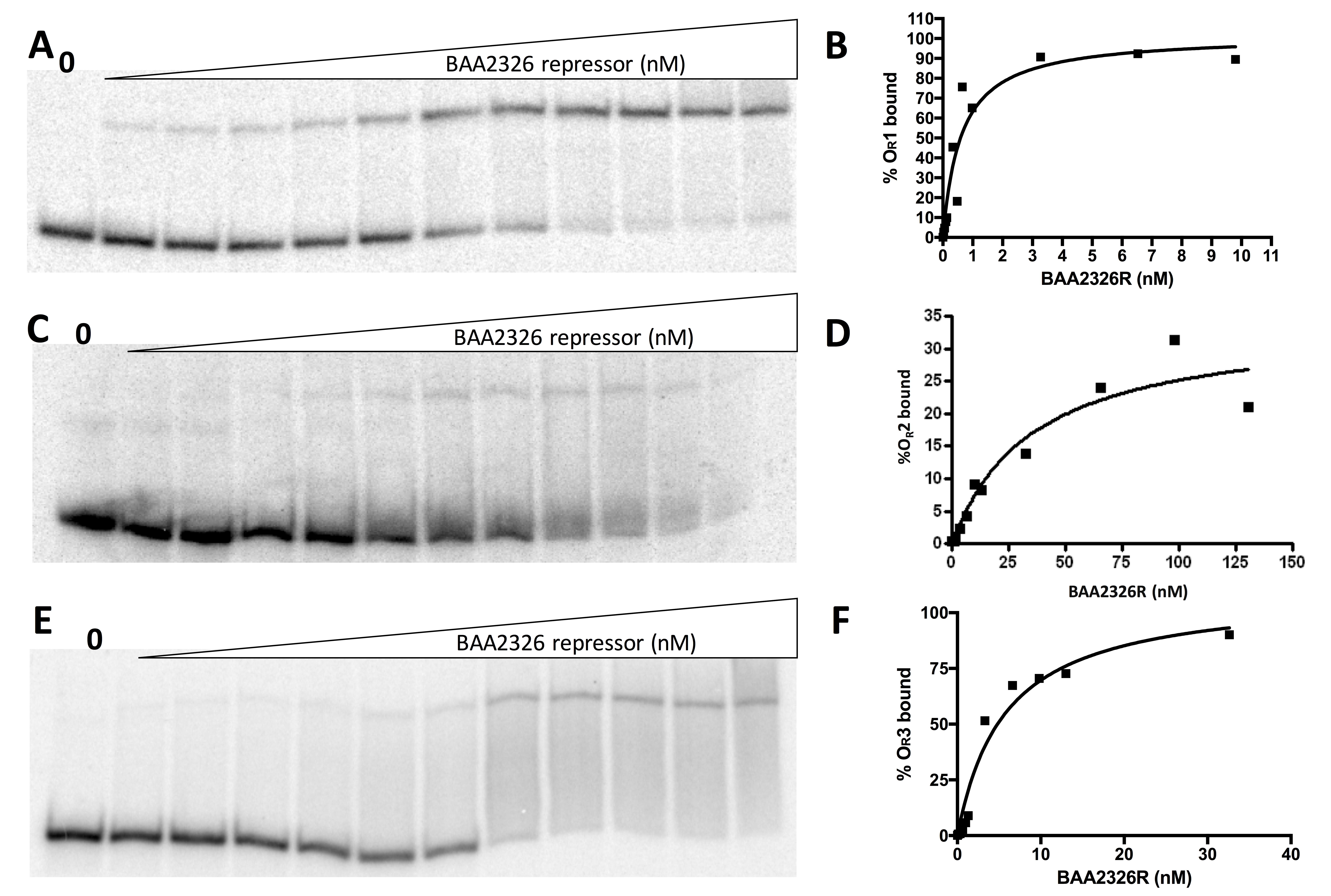

Supplement: Supplementary file 1 [file viruses-10-00228-s001.zip › fiinal supplemental/Figure_S2-Final.tiff]

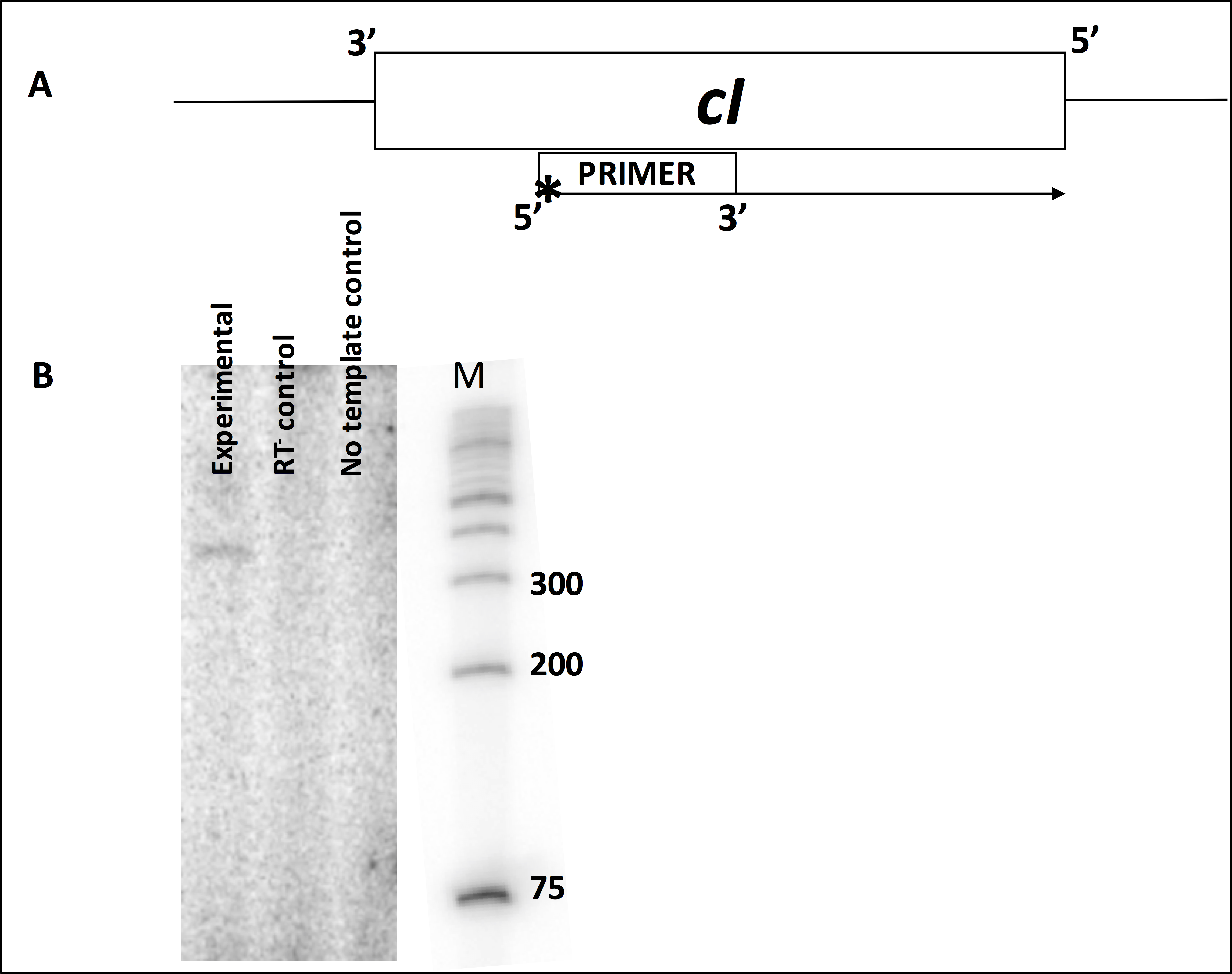

Supplement: Supplementary file 1 [file viruses-10-00228-s001.zip › fiinal supplemental/Figure_S3-Final.tiff]

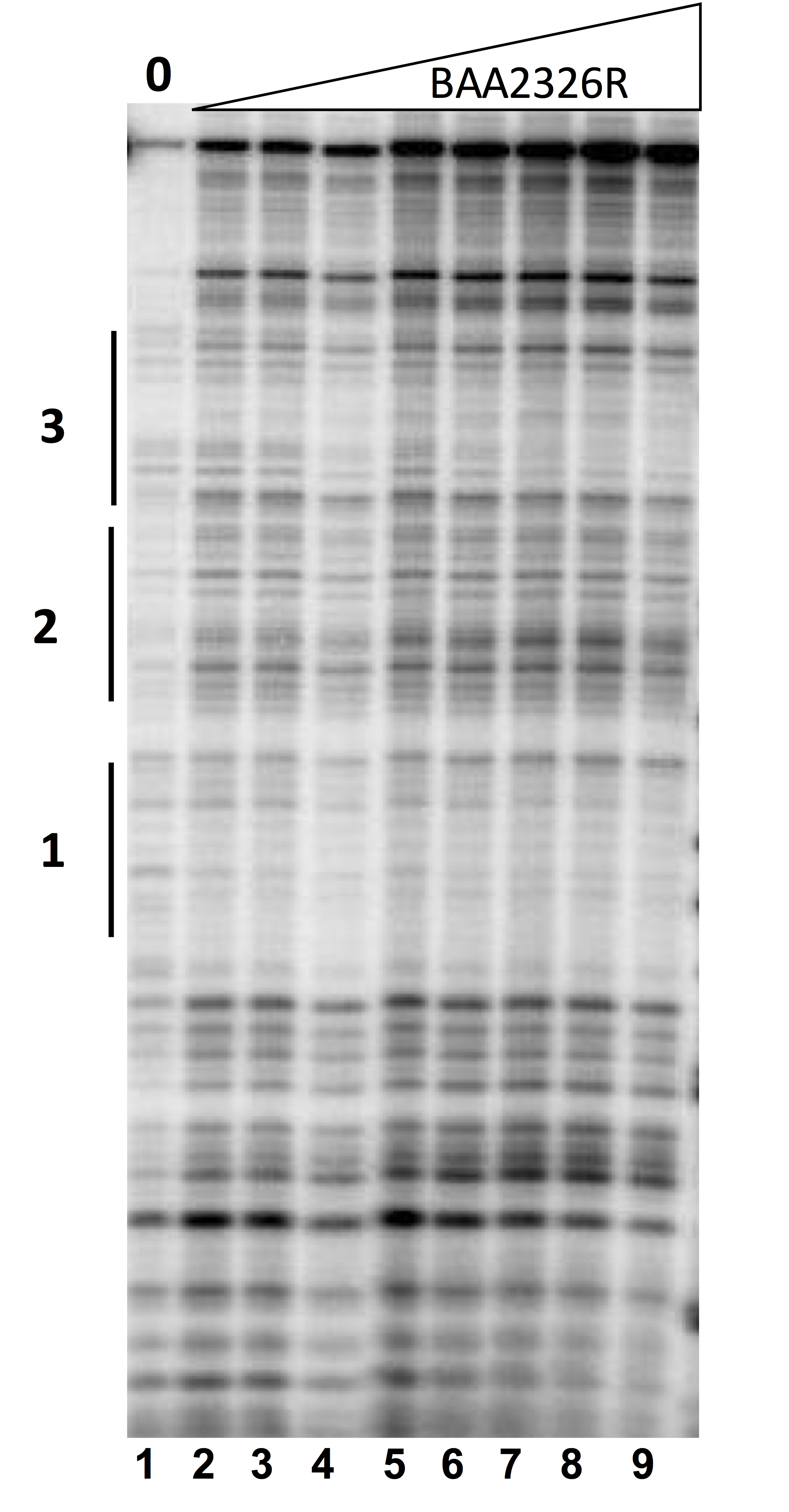

Supplement: Supplementary file 1 [file viruses-10-00228-s001.zip › fiinal supplemental/Figure_S4-Final.tiff]
